# Editorial comment to “Utility of cardiac implantable electronic device algorithm for detecting severe sleep‐disordered breathing in cardiomyopathy”

**DOI:** 10.1002/joa3.13167

**Published:** 2024-11-01

**Authors:** Mitsuharu Kawamura

**Affiliations:** ^1^ Makita General Hospital Tokyo Japan

In this issue of the *Journal of Arrhythmia*, Li et al.^1^ described the relationship between severe sleep‐disordered breathing (SDB) and transthoracic impedance sensing to calculate respiratory disturbance index (RDI) in patients with implantable cardiac defibrillator (ICD). Severe SDB was defined as polysomnography (PSG)–apnea–hypopnea index (AHI) ≥30 episodes/h and was diagnosed by PSG in 66.7% of the patients. RDI was found to have a positive correlation with PSG‐AHI (*r* = .473, *p* = .027). Using the cutoff threshold of RDI for screening of severe SDB of 32 produces a sensitivity of 91.7% and specificity of 16.7%. RDI of 48 episodes/h demonstrated a specificity of 100% and a sensitivity of 58.3% for severe SDB. Defaye et al.^2^ reported a prevalence of 78% in moderate to severe SDB and 56% in severe SDB in patients with pacemakers. An optimal cutoff of 20 events/h for the sleep apnea monitoring RDI value was validated to identify severe SDB with a sensitivity of 88.9%, a positive predictive value of 88.9%, and a specificity of 84.6%. SDB is highly prevalent in patients with cardiovascular diseases including heart failure. A transthoracic impedance sensor with an advanced algorithm, the sleep apnea monitoring algorithm, could be used to identify severe SDB in patients with pacemakers. Khayat et al.^3^ supported that over 80% of patients with heart failure may have SDB and these patients confers significant disease burden, as well as high morbidity. A recent meta‐analysis by Messaoud et al.^4^ assessing the utility of all ICDs (pacemakers and ICDs) revealed that among the 16 included studies, only four examined patients with implanted ICDs, all within Caucasian populations and indicated that the sensitivity of cardiac implants for SDB diagnosis ranged from 60% to 100%, with specificity from 50% to 100%.

Chen et al.^5^ reported 64 patients were enrolled, who had never been diagnosed with SDB or underwent PSG examination. After PSG examination, 76.4% patients were diagnosed as combining with SDB (20% severe, 18.2% moderate, and 38.2% mild). RDI calculated by pace markers has a strong positive correlation with PSG‐AHI (*r* = .76, *p* < .001, 95% CI 0.61–0.85). The optimal cutoff value of PM‐RDI for advanced SDB (PSG‐AHI ≥15) diagnosis was 26, with area under the curve of 0.89. The best cutoff value for severe SDB (PSG‐AHI ≥30) identification was 41, with a sensitivity of 81.6%, a specificity of 88.6%. Pacemaker patients present a high prevalence of undiagnosed SDB. Detection of SDB by pacemaker is feasible and accurate in SDB screening and monitoring.

In this issue, Li et al.^1^ studied 18 patients completed PSG study and all patients had an ICD. The ICD algorithm uses transthoracic impedance sensing to calculate RDI. Twelve patients (66.7%) had ICD implanted for primary prevention. The Etiology of cardiomyopathy was ischemic in 15/18 (83.3%) and due to channelopathy in 1 (0.55%) of patients. Li et al.^1^ included patients with obstructive sleep apnea (OSA), central sleep apnea (CSA), and mixed sleep apnea (MSA). The distribution of this small cohort consisted of 15 patients with OSA, 1 with CSA, and 2 with MSA. The authors did not choose to exclude CSA or MSA patients despite the different underlying mechanisms, as this primary aim was to gather preliminary data and assess the feasibility of the study across a spectrum of sleep apnea types. I am impressed this issue, because this study has evaluated the algorithm in ICD, in a multi‐ethnic Asian population with heart failure. The authors that applying a binary threshold cutoff may be useful in detecting severe SDB with high sensitivity in this group of ICD patients. SDB is prevalent in patients with heart failure. However, it is underdiagnosed as these patients seldom present with typical symptoms of heart failure. Undiagnosed severe SDB poses increased morbidity and mortality to an already vulnerable group of patients. In this issue, transthoracic impedance sensing with an advanced inbuilt algorithm emerged as a promising approach for screening of SDB and identified a separate threshold for detecting severe SDB among patients with heart failure and cardiomyopathy. This study represents one of the earliest validations of the algorithm in an exclusively multi‐ethnic Asian population with heart failure, marking a significant contribution to the field. However, this study had some limitations and problems. This study is the small size of the cohort. Only 18 patients participated in this study. I feel it is difficult to make a conclusion with this small number of patients. Furthermore, the authors included CSA and MSA in this study. I feel these sleep apnea mechanisms are different from OSA. Therefore, further studies with a larger patient cohort across multiple centers are needed to clarify the relationship between severe SDB and transthoracic impedance sensing to calculate RDI in patients with ICD. We need to evaluate relationship between moderate and mild SDB, and transthoracic impedance sensing to calculate RDI in patients with ICD. Furthermore, we need to investigate the RDI in patients with severe SDB treated with continuous positive airway pressure before and after. Finally, future advancements in technology will enable more precise differentiation of sleep apnea types, enhancing the accuracy and utility of non‐PSG screening tools.

## CONFLICT OF INTEREST STATEMENT

Authors declare no conflict of interests for this article.
